# Nurse Staffing Configurations and Nurse Absence Due to Sickness

**DOI:** 10.1001/jamanetworkopen.2025.5946

**Published:** 2025-04-22

**Authors:** Chiara Dall’Ora, Paul Meredith, Christina Saville, Jeremy Jones, Peter Griffiths

**Affiliations:** 1 School of Health Sciences, University of Southampton, Southampton, England, United Kingdom; 2 National Institute for Health Research Applied Research Centre, Wessex, Southampton, England, United Kingdom

## Abstract

**Importance:**

Rates of work-related stress and sickness absence are high among nurses. The consequences of sickness absence are severe for health systems’ efficiency and productivity.

**Objective:**

To examine the association between nurse staffing configurations and sickness absence in hospital inpatient unit nursing teams.

**Design, Setting, and Participants:**

This retrospective longitudinal case-control study using routinely collected hospital data among 4 general acute care hospitals in England examined 18 674 registered nurses (RNs) and nursing support (NS) staff working in 116 hospital units. Data were analyzed from April 1, 2015, to February 29, 2020.

**Exposure:**

Nursing team skill mix, temporary staffing hours, understaffing, proportion of long shifts (≥12 hours) worked, and full-time or part-time work status in the previous 7 days.

**Main Outcome and Measure:**

The main outcome was the number of episodes of sickness absence, which was defined as a sequence of sickness days with no intervening days of work.

**Results:**

There were 2 690 080 shifts and 43 097 sickness episodes. The incident rates for starting a sickness episode were 2.0% for NSs and 1.4% for RNs. In total, there were 18 674 members of staff; 2 188 562 (81.6%) were shifts by staff classified as working full time and 493 400 (18.4%) by staff classified as working part time. A skill mix composed of more RNs was associated with a 2% reduction in the odds of RN sickness absence for each 10% change in the proportion of RN hours (odds ratio [OR], 0.98; 95% CI, 0.96-0.99). Moving from the lower quartile skill mix (ie, approximately 55% of the nursing hours worked by RNs) to the upper quartile (ie, approximately 75% of the nursing hours worked by RNs) was associated with a 4% reduction in odds of sickness absence. Working only long shifts in the prior 7 days was associated with a 26% increase in the odds of sickness absence for RNs compared with working no long shifts (OR, 1.26; 95% CI, 1.19-1.33). Part-time work for RNs was associated with higher sickness absence (OR, 1.09; 95% CI, 1.03-1.15). For RNs, there was a significant interaction between part-time work and RN understaffing, whereby short staffing in the previous week increased sickness absence for full-time staff but not among those working part time. NS understaffing was not associated with sickness absence for any staffing group.

**Conclusions and Relevance:**

This retrospective case-control study found that exposure to RN understaffing increased the risk of sickness absence in nursing teams. Adverse working conditions for nurses, already known to pose a risk to patient safety, may also create risks for nurses and the possibility of further exacerbating staff shortages.

## Key Points

**Question:**

What is the association between variation in nurse staffing configurations and nurses’ sickness absence?

**Findings:**

In this longitudinal case-control study of 18 674 registered nurses (RNs) and nursing support (NS) staff, RN understaffing in the preceding 7 days was associated with sickness absence for NS staff; however, for RNs, the association was observed only when working full time. A skill mix composed of more RNs and working no long shifts (ie, ≥12 hours) in the preceding 7 days were protective factors against RN sickness absence.

**Meaning:**

These findings suggest that, to support nurses’ health and health systems’ productivity and efficiency, investing in avoiding RN understaffing may be warranted.

## Teaser

This case-control study examines the association between nurse staffing configurations and sickness absence in hospital inpatient unit nursing teams in England.

## Introduction

The nursing workforce worldwide is experiencing high levels of work-related stress and absence due to sickness (referred to as *sickness absence* from here onward), with concerns regarding nurses’ ability to cope with such high pressure in the long term.^1^ Every year, England’s National Health Service (NHS) nursing staff report considerable dissatisfaction with their jobs and feelings of burnout,^2^ with levels of sickness absence remaining higher than those of most other health professions.^3^

The implications of sickness absence levels are serious both in terms of lost productivity and increased costs to employers and society.^4^ Mental health–related reports, including stress and anxiety, are the main cause of sickness absence in nursing staff in England, making up around one-fourth of all absences.^3^ In the NHS staff survey, nearly half of the nurses reported that they felt unwell because of work-related stress in the last 12 months.^2^

Although sickness absence is a complex phenomenon and not always caused by occupational factors, modifiable work-related factors, including long shifts, are associated with nurses’ sickness absence.^5,6^ Nurse staffing levels have been associated with a range of staff outcomes in cross-sectional survey studies, including burnout, job dissatisfaction, and turnover intention,^7,8^ but the effect on sickness absence rates has not been studied directly. Similarly, a lower registered nurse (RN) skill mix (ie, lower proportions of RNs within the nursing team configuration) and working on shifts with higher levels of temporary bank and agency staff have been associated with increased turnover^9^ and job dissatisfaction,^10^ but the effect on sickness absence is unknown.

This knowledge gap is likely to have originated from unavailability of objective sickness absence data, but advances in research using data extracted from routinely collected electronic hospital systems mean that this gap can now be addressed. Therefore, the aim of this study was to examine the association between nurse staffing configurations and nurse sickness absence in general acute care hospitals.

## Methods

This was a retrospective longitudinal case-control study in 4 NHS hospital trusts across England. These trusts display variation in terms of geographical location (ie, London, Southeast, Southwest, and Midlands), urbanity or rurality (ie, 3 hospitals are urban, and 1 is rural), and levels of affluence and deprivation. They also exhibit variation in trust type (2 are teaching hospitals), size (523-1746 beds), and average RN staffing levels (1.38-2.74 RNs per occupied bed). We included all adult acute care inpatient units (including high-dependency units and intensive care units) and excluded maternity and pediatric units. This study received ethical approval by the Health Research Authority and the University of Southampton ethics committee. This report adheres to the Strengthening the Reporting of Observational Studies in Epidemiology (STROBE) reporting guideline for case-control studies.

Our data sources were electronic rostering systems containing records of all nursing staff rostered to work on a unit on a given shift, records of temporary bank (ie, hospital-employed staff working extra hours beyond their contract) and external agency staff working on the unit, and the hospitals’ patient administration systems. We used the latter to quantify how many patients were on a unit at any given point in time. Data were available from April 2015 to February 2020 in most trusts except for 1 case in which the data were available from March 2019. We extracted 3 583 586 shifts from 123 units. Informed consent was not required because the data were extracted by routinely collected administrative datasets; staff identifiers in rostering records were pseudonymized, allowing the linkage of shifts worked by the same individual across the study period, including their sickness absences. Sickness absence data were available for employed staff only. Shifts that were not worked due to nursing staff sickness were aggregated into sickness episodes, starting on the first day that a nurse was absent from work and finishing on the day they returned for at least 1 shift. Sickness was treated as a binary categorical variable occurring on the first day of an episode of sickness. We removed episodes that were not preceded by any worked hours in the previous 7 days.

We calculated unit staffing levels for 12-hour shift periods starting at 7 am (day) and 7 pm (night) by aggregating rostered nursing hours of care, including temporary assignments to the unit (ie, bank and agency). We calculated staffing hours excluding breaks by assuming breaks were taken in the middle of shifts. We then linked the nursing hours to the patient occupancy on the unit for the same 12-hour periods. This was achieved by calculating the occupancy for each patient on the unit during the shift using the admission, discharge, and transfer information for the unit, aggregating the occupancy durations and then converting the total occupancy duration into a number of patient-days. We calculated hours per patient-day (HPPD) for each shift as the sum of hours worked by each staff group divided by the number of patient-days. We calculated RN and nursing support (NS) staffing levels separately using the NHS Agenda for Change pay bands to identify RNs (bands ≥5) and NSs (bands 2-4). NSs work under the guidance of RNs and support them in the delivery of nursing services. We defined an expected staffing level by shift period (day or night) for each staff group on each unit by calculating the mean staffing, with the expected staffing rebased when there were clear discontinuities in unit use, identified by substantial changes in patient case mix. Rebasing involved calculating unit staffing means for each consistent period of case mix around a discontinuity. Shifts with unfeasibly low (<11 hours of RN staffing in a 12-hour period or <0.5 RN HPPD) or high (>48 RN HPPD) staffing were excluded, and they were assumed to be representative of a mismatch between patient and staff records occurring during periods of unit reorganization.

For each staff member, we calculated various exposure measures to staffing configurations for all shifts worked in the past 7 days. These exposure measures were understaffing of RN and NS staff members, the proportion of staff hours provided by temporary bank and agency staff members on the unit, the skill mix, and whether the nurse worked only long shifts (ie, shifts ≥12 hours). In England’s NHS, a nursing shift length of 12.5 hours is the norm, but variation exists, and some individuals opt to work shorter shifts. Understaffing was calculated as the mean of 1 − (observed HPPD/expected HPPD), where the observed HPPD was smaller than expected (ie, as a unidirectional measure). Skill mix was calculated as the mean proportion of RN hours over all of the nursing hours on the unit for all shifts worked in the previous week. We did not have access to data on employees’ full-time or part-time status, so we classified an employee as part time when their median number of weekly worked hours in the previous 13 weeks was less than or equal to 26 hours.

### Statistical Analysis

We examined the association between staffing configurations and sickness absence with generalized linear mixed models with a logit link function. We calculated intraclass correlation coefficients from unconditional random intercept models to assess the within-unit and the within-staff variation for sickness. There was variation in sickness episodes at the individual level (intraclass correlation coefficient, 0.85) and at the unit level (intraclass correlation coefficient, 0.49), so both were included as random effects. All analyses were performed at the shift level. Because sickness absence rates differ substantially for RNs and NSs, we modeled them separately.^3^ To aid interpretations of results, we input understaffing, bank and agency, and skill mix as 10–percentage point increments in our models. This means, for example, that if the mean level of understaffing for each worked shift in the previous week was 10%, the mean staffing level for the unit in the previous week was 90% of the expected staffing level. For bank or agency hours, this refers to their proportion relative to the total nursing hours for all shifts worked in the previous week. For instance, if the total number of nursing hours in the unit was 300, a 10% bank or agency proportion would mean 30 of those hours were covered by bank or agency staff. Because long shifts are the norm in England’s NHS, we report the point estimates with working no long shifts as the reference category vs working all long shifts in the previous week. We estimated univariable models (single staffing factor), a full model (all staffing factors), and a parsimonious reduced model using backward stepwise selection, removing the variables with the highest *P* value at each step, provided this would lead to reductions in the Akaike information criterion and the bayesian information criterion. In the backward stepwise selection, we did not remove RN understaffing because it was a theoretical focal point of our analysis. Because the effect of RN understaffing on sickness absence might depend on other variables, including NS understaffing, bank and agency hours, and skill mix, we tested for interactions between RN understaffing and these variables. To exclude multicollinearity, we checked the variance inflation factor of all models; variance inflation factor scores were lower than 2, indicating low multicollinearity.^11^ Data analyses were undertaken using R, version 4.2.2 (R Project for Statistical Computing)^12^ and the lme4 package.^13^ *P* values relate to the *B* coefficients of the regression models. Data were considered significant with a 2-sided *P* < .05. Data were analyzed from April 1, 2015, to February 29, 2020.

## Results

After removing all shifts with unrealistic staffing levels (2.2%) and shifts worked in units that were not part of our analysis (ie, maternity and pediatrics), our sample was 2 690 080 shifts in 116 units, of which 43 097 were the first day of a sickness episode. The incident rates for starting a sickness episode were 2.0% for NSs and 1.4% for RNs. In total, there were 18 674 members of staff; 2 188 562 (81.6%) were shifts by staff classified as working full time, and 493 400 (18.4%) by staff classified as working part time.

Descriptive statistics for exposure to staffing configurations in the past week by sickness cohort are reported in Table 1. All odds ratios (ORs) and 95% CIs of associations between staffing configurations and sickness absence are reported in Table 2.

**Table 1.** Staffing Configurations by Sickness Cohort

Exposure in the past week	All groups, %		RNs, %		NS staff members, %	
	Sick	Worked	Sick	Worked	Sick	Worked
RN understaffing						
Median (IQR)	5.5 (0-13.8)	5.3 (0-13.3)	4.7 (0-12.4)	4.7 (0-12.1)	6.6 (0-16.0)	6.6 (0-15.6)
Mean (SD)	9.1 (10.8)	8.7 (10.3)	8.1 (10.0)	7.8 (9.7)	10.3 (11.7)	10.1 (11.4)
NS understaffing						
Median (IQR)	7.9 (0-20.4)	8.3 (0-21.1)	9.9 (0-23.1)	9.9 (0-23.2)	5.5 (0-16.7)	5.5 (0-16.7)
Mean (SD)	13.3 (16.2)	13.7 (16.5)	15.0 (17.3)	15.2 (17.3)	10.8 (14.2)	10.9 (14.3)
Agency staffing (proportion)						
Median (IQR)	0.0 (0-3.6)	0.0 (0-2.6)	0.0 (0-2.6)	0.0 (0-1.6)	0.0 (0-4.7)	0.0 (0-4.2)
Mean (SD)	3.0 (6.1)	2.5 (5.4)	2.6 (5.6)	2.2 (4.9)	3.5 (6.8)	3.2 (6.2)
Bank staffing (proportion)						
Median (IQR)	0.0 (0-12.9)	0.0 (0-11.7)	0.0 (0-12.1)	0.0 (0-10.6)	0.0 (0-13.8)	0.0 (0-13.6)
Mean (SD)	7.3 (10.6)	6.7 (10.2)	7.0 (10.6)	6.3 (9.9)	7.8 (10.6)	7.6 (10.5)
Skill mix (proportion)						
Median (IQR)	61.6 (51.0-72.6)	63.5 (52.5-75.0)	66.7 (56.2-79.0)	68.0 (57.1-80.8)	55.5 (47.1-64.8)	55.6 (47.1-65.2)
Mean (SD)	62.9 (15.7)	64.7 (16.1)	68.0 (15.8)	69.0 (15.9)	56.3 (12.7)	56.4 (13.1)
Long (≥12 h) shifts (proportion)						
Median (IQR)	100.0 (66.7-100.0)	100.0 (66.7-100.0)	100.0 (66.7-100.0)	100.0 (66.7-100.0)	100.0 (50.0-100.0)	100.0 (66.7-100.0)
Mean (SD)	79.2 (34.6)	78.0 (35.8)	81.2 (33.2)	78.1 (35.6)	76.5 (36.3)	77.6 (36.3)

Abbreviations: NS, nursing support; RN, registered nurse.

**Table 2.** Regression: Univariable and Multivariable Associations Between Staffing Configurations and Sickness Absence^a^

Variable	Univariable associations		Full model		Parsimonious reduced model	
	OR (95% CI)	*P* value	OR (95% CI)	*P* value	OR (95% CI)	*P* value
**RN sickness absence^b^**						
Exposure in the preceding 7 d						
RN understaffing	1.02 (1.01-1.03)^c^	.009	1.01 (0.99-1.02)	.58	1.01 (0.99-1.03)	.15
NS understaffing	1.00 (0.99-1.01)	.95	1.00 (0.99-1.01)	.68	NA	NA
Bank hours	0.98 (0.96-0.99)^c^	.03	0.95 (0.93-0.97)^c^	.001	0.95 (0.94-0.97)^c^	<.001
Agency hours	1.02 (0.99-1.05)	.15	1.02 (0.99-1.05)	.26	NA	NA
Skill mix	0.99 (0.97-1.00)	.13	0.98 (0.96-1.00)	.11	0.98 (0.96-0.99)^c^	.04
Work patterns in the preceding 7 d						
All long (≥12 h) shifts (vs no long shifts)	1.29 (1.22-1.35)^c^	<.001	1.26 (1.19-1.33)^c^	.001	1.26 (1.19-1.33)^c^	<.001
Part time (no vs yes)	1.03 (0.99-1.08)	.14	1.05 (1.01-1.09)^c^	.03	1.09 (1.03-1.15)^c^	.002
Interaction: RN understaffing × part time	NA	NA	NA	NA	0.96 (0.92-0.99)^c^	.01
**NS sickness absence^d^**						
Exposure in the preceding 7 d						
RN understaffing	1.03 (1.01-1.03)^c^	<.001	1.03 (1.01-1.04)^c^	.005	1.02 (1.00-1.03)^c^	.02
NS understaffing	1.01 (0.99-1.02)	.31	0.99 (0.98-1.01)	.29	1.00 (0.98-1.01)	.76
Bank hours	0.95 (0.93-0.97)^c^	<.001	0.92 (0.90-0.94)^c^	.001	0.92 (0.90-0.94)^c^	<.001
Agency hours	1.01 (0.99-1.04)	.32	1.03 (1.00-1.06)	.07	1.03 (1.00-1.06)	.07
Skill mix	1.01 (0.99-1.03)	.34	1.02 (1.00-1.05)	.09	NA	NA
Work patterns in the preceding 7 d						
All long (≥12 h) shifts (vs no long shifts)	1.03 (0.97-1.10)	.24	0.98 (0.92-1.04)	.52	NA	NA
Part time (no vs yes)	1.04 (0.91-0.95)	.10	1.04 (0.99-1.09)	.11	1.04 (0.99-1.10)	.10

Abbreviations: NA, not applicable; NS, nursing support; OR, odds ratio; RN, registered nurse.

^a^ All models include staff identification and ward as random effects.

^b^ The Akaike information criterion is 219 942 for the full model and 219 935.2 for the parsimonious reduced model; the bayesian information criterion is 220 065.8 for the full model and 220 046.7 for the parsimonious reduced model.

^c^ Significant at *P* < .05.

^d^ The Akaike information criterion is 143 998.4 for the full model and 143 998.1 for the parsimonious reduced model; the bayesian information criterion is 144 115.6 for the full model and 144 091.9 for the parsimonious reduced model.

**Figure 1.**
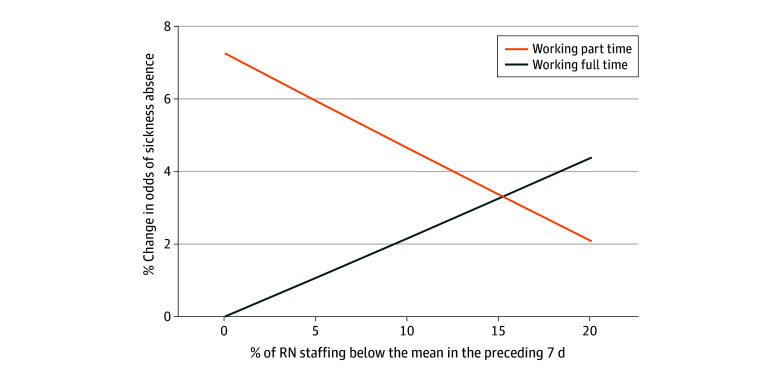
Interaction Between Registered Nurse (RN) Understaffing and Part-Time Work: Change in Odds Ratios Change in the odds of sickness absence associated with variation in staffing levels relative to the mean by part-time or full-time status.

In our reduced parsimonious model considering RN sickness absence, working only long shifts in the prior 7 days was associated with a 26% increase in the odds of sickness absence (OR, 1.26; 95% CI, 1.19-1.33) compared with working no long shifts. For a 10–percentage point increase in exposure to bank hours in the previous week, there was a 5% reduction in the odds of sickness absence (OR, 0.95; 95% CI, 0.94-0.97). Skill mixes composed of more RNs were associated with lower sickness absence (exposure to a 10% higher skill mix: OR, 0.98; 95% CI, 0.96-0.99). Working part time was associated with higher sickness absence (OR, 1.09; 95% CI, 1.03-1.15). Although the association between RN understaffing and RN sickness absence was not statistically significant in the reduced parsimonious model, the OR was in the hypothesized direction (OR, 1.01; 95% CI, 0.99-1.03). We found a statistically significant interaction (OR, 0.96; 95% CI, 0.92-0.99;*P* = .01) between exposure to RN understaffing and working part time. To understand the interaction, we plotted the curves based on the *B* coefficients of RN understaffing, part-time work, and their interaction (Figure 1).

**Figure 2.**
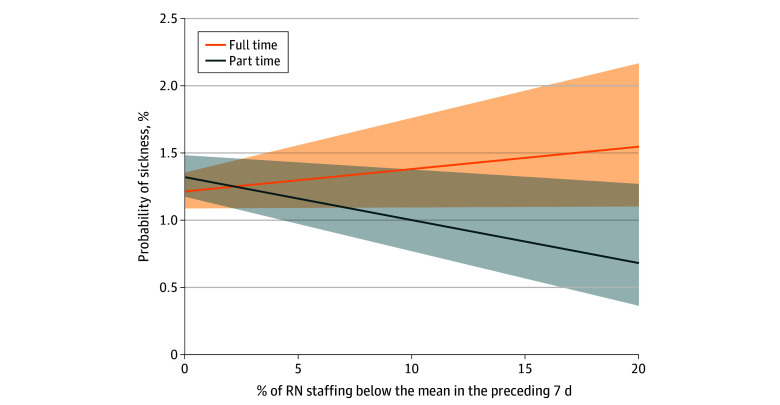
Interaction Between Registered Nurse (RN) Understaffing and Part-Time Work: Marginal Probabilities Marginal probabilities of sickness absence associated with variation in staffing levels. Shaded areas indicate 95% CIs.

The odds of sickness absence were increased when RNs were exposed to higher proportions of RN understaffing and working full time, whereas working part time reverses the association between RN understaffing and sickness absence. All other interactions that we tested for were not statistically significant. In absolute terms, the marginal probability for a full-time RN who has been exposed to a 10–percentage point increase in exposure to RN understaffing is 51% (Figure 2). This indicates a slight increase in probability. For NSs in the parsimonious reduced model, only exposure to RN understaffing (for every 10–percentage point increase in RN understaffing: OR, 1.02; 95% CI, 1.00-1.03) and bank hours (for every 10–percentage point increase in exposure to bank hours on the ward: OR, 0.92; 95% CI, 0.90-0.94) in the past week were predictors of sickness absence.

To aid interpretation of our findings, we put them into context. For example, increasing the RN skill mix by 10% would lead to a 2% reduction in RN sickness absence. The mean number of RN shifts for each hospital is 111 000 per year. An RN sickness absence rate of 1.4% means that every year, the mean number of RN sickness episodes is 1565 per hospital. The mean duration of each RN sickness episode was 12 days, so there are 18 780 days lost due to RN sickness each year. Reducing sickness absence by 2% would lead to 376 fewer days lost to sickness. A hospital with an upper-quartile skill mix (ie, approximately 75% of the nursing hours are worked by RNs) will have 4% less sickness absence, equating to 752 fewer days lost to sickness absence per year compared with a hospital in the lower-quartile skill mix (ie, approximately 55% of the nursing hours worked by RNs).

## Discussion

To our knowledge, this was the first study to analyze the association between nurse staffing configurations and nurses’ sickness absence using objective data extracted from hospital systems. With data collected over 5 years in 4 hospitals, we uncovered statistically significant associations between a number of staffing variables and sickness absence. Our study revealed 4 key findings. First, we found that RN understaffing in the preceding 7 days was associated with sickness absence for NSs but that for RNs the association was seen only in those working full time. Second, we found being exposed to higher proportions of hours worked by bank nursing staff was associated with a lower number of sickness absences for RNs and NSs. Third, RNs working shifts with a skill mix composed of more RNs in the preceding 7 days were less likely to experience sickness absence. Last, RNs working higher proportions of long shifts in the preceding period were more likely to experience sickness absence. These findings are significant because the nursing workforce globally is under increasing pressure, with higher proportions of RNs experiencing stress-related sickness and leaving their jobs as a consequence and with not enough RNs entering the workforce.^14,15^ Our results contribute to the list of known harms of RN understaffing and diluted skill mixes that have currently mainly focused on patient outcomes.^16,17^ Although the absolute effects of increasing the RN skill mix and reducing RN understaffing might seem small, in a climate where achieving staffing targets remains challenging with serious consequences for patient safety, even small improvements in working conditions are likely to lead to improved productivity and reduced turnover. This has been demonstrated in previous research using purely subjective measures.^10,18^ Staff working part time (18.4% of our sample shifts were staffed by nurses classified as working part time) do not appear to experience the adverse effects of low RN staffing; indeed, low staffing appears to reduce their risk of sickness. This may in part be an artifact because these staff members will be working fewer days, so the variable expressed as a proportion relates to fewer days than it does for full-time staff. The finding that part-time work is associated with a higher sickness absence rate is novel and might stem from the healthy worker effect, whereby workers who are fit and healthy are likely to work more hours.^19^ A form of healthy worker effect may also explain the counterintuitive decrease in sickness absences when exposed to understaffing for this group because, if healthy, they also have more capacity to work increased hours when staffing is challenged.

RN understaffing and diluted skill mixes might lead to increased pressure and stress, higher levels of burnout, and lower job satisfaction within nursing staff^20,21^ because RNs are responsible for a number of complex activities that cannot be delegated to NSs. Deploying enough or more NSs to counter RN understaffing does not appear to be an effective solution to relieve the pressure on the nursing team. Higher nursing staff stress and pressure might act as mediators in the relationship between RN understaffing and sickness absence, creating a negative vicious circle in which staff well-being decreases and sickness absence escalates. Increasing the proportion of NSs might lead to capacity issues and more stress and pressure on RNs because they cannot adequately support and supervise NSs, meaning that the assumed benefits from increasing overall nursing numbers are not realized and the RN workforce is negatively affected. This is an important finding at a time when challenges to employing enough RNs are driving the deployment of a more diluted skill mix.^22^

Relatedly, we did not find any evidence of adverse effects from low NS staffing that mirrors the effects from RN staffing, emphasizing that low RN staffing levels are the central problem that should be addressed. In the face of low NS staffing, RNs can flex their role to cover gaps, whereas NSs are unable to do the same to cover for RN shortages.

Although the high use of temporary staff has been associated with adverse outcomes for staff and patients in previous research,^23,24^ we did not see clear evidence of any such effect. It is possible that high use of these temporary staff results from successful attempts to manage understaffing, although we did not observe an interaction between the 2 variables. In view of previous findings, this warrants further study.

When RNs worked high proportions of long shifts of 12 or more hours, they were more likely to experience sickness absence. This has been observed previously.^5,6^ Our study strengthens previous findings thanks to its larger and more diverse sample. It also corroborates the hypothetical mechanism whereby long shifts lead to higher cumulative fatigue,^25^ meaning that the resulting extra days off do not constitute an adequate recovery mechanism, and staff experience sickness absence as a result.

### Limitations

This study has limitations. Although our study is longitudinal and we consider a number of working conditions to which staff are exposed before experiencing sickness absence, the negative findings around RN staffing variables might be reflective of poor working environments (eg, a good unit work environment has simultaneously better RN staffing levels and fewer long shifts and sickness levels), but our data do not allow us to disentangle this. Nonetheless, our findings are similar to those of previous studies^7,26^ in which poor staffing configurations were consistently associated with negative outcomes for patients and staff. Sickness absence can cause understaffing, a dilution in skill mix, and higher use of agency staff, so these variables could be correlated. However, we tested for interactions and correlations and did not find evidence of any significant ones.

Although sickness absence is an objective indicator of staff behavior, it is multifactorial in nature, and we did not have access to the main reason for sickness absence or any other demographics that could influence an employee’s likelihood of experiencing sickness absence. Nonetheless, we were able to cluster sickness absence episodes within individuals, meaning that personal characteristics were partially controlled for.

## Conclusions

In this longitudinal case-control study, when a clustering of adverse staffing configurations—low RN staffing levels, skill mixes containing fewer RNs, and working long shifts—occurred, the consequences for nursing staff were severe, and their odds of experiencing sickness absence increased. In a climate in which workforce well-being, efficiency, and productivity are high on health systems’ agendas, those in charge of planning a workforce at strategic and local levels should invest in employing more RNs in hospital settings. Increasing RN staffing levels has been associated with improved outcomes for patients and reduced costs,^27^ and our study adds to this body of evidence by shining a light on the additional effect on nurse health and well-being. Given the considerable costs associated with sickness absence, investing in RNs has the potential to reduce the pressure on an already exhausted workforce and health system.

## Notes

Supplement.
Data Sharing Statement
