# Supplementary material for: Nurse Staffing Configurations and Nurse Absence Due to Sickness
Source: JAMA Netw Open. 2025 Apr 22;8(4):e255946. doi: 10.1001/jamanetworkopen.2025.5946 (PMC12015667; doi:10.1001/jamanetworkopen.2025.5946)
Supplement: Supplement. — Data Sharing Statement [file jamanetwopen-e255946-s001.pdf]

Data Sharing Statement

Dall'Ora. Nurse Staffing Configurations and Nurse Absence Due to Sickness. *JAMA Netw Open*. Published April 22, 2025. doi:10.1001/jamanetworkopen.2025.5946

Data
Data available: No

Additional Information
Explanation for why data not available: No data can be shared. Unfortunately, due to the sensitive nature of the data and data sharing agreements with the providers, we are unable to freely share the source data, but we guarantee its authenticity and the rigour of methods used in the analysis.
